# Supplementary figures and images for: Association between Practising Religion and Cardiovascular Disease Risk among Ghanaian Non-Migrants and Migrants in Europe: The RODAM Study
Source: Int J Environ Res Public Health. 2021 Mar 2;18(5):2451. doi: 10.3390/ijerph18052451 (PMC7967581; doi:10.3390/ijerph18052451)

**S1. Study participation by site (n).**

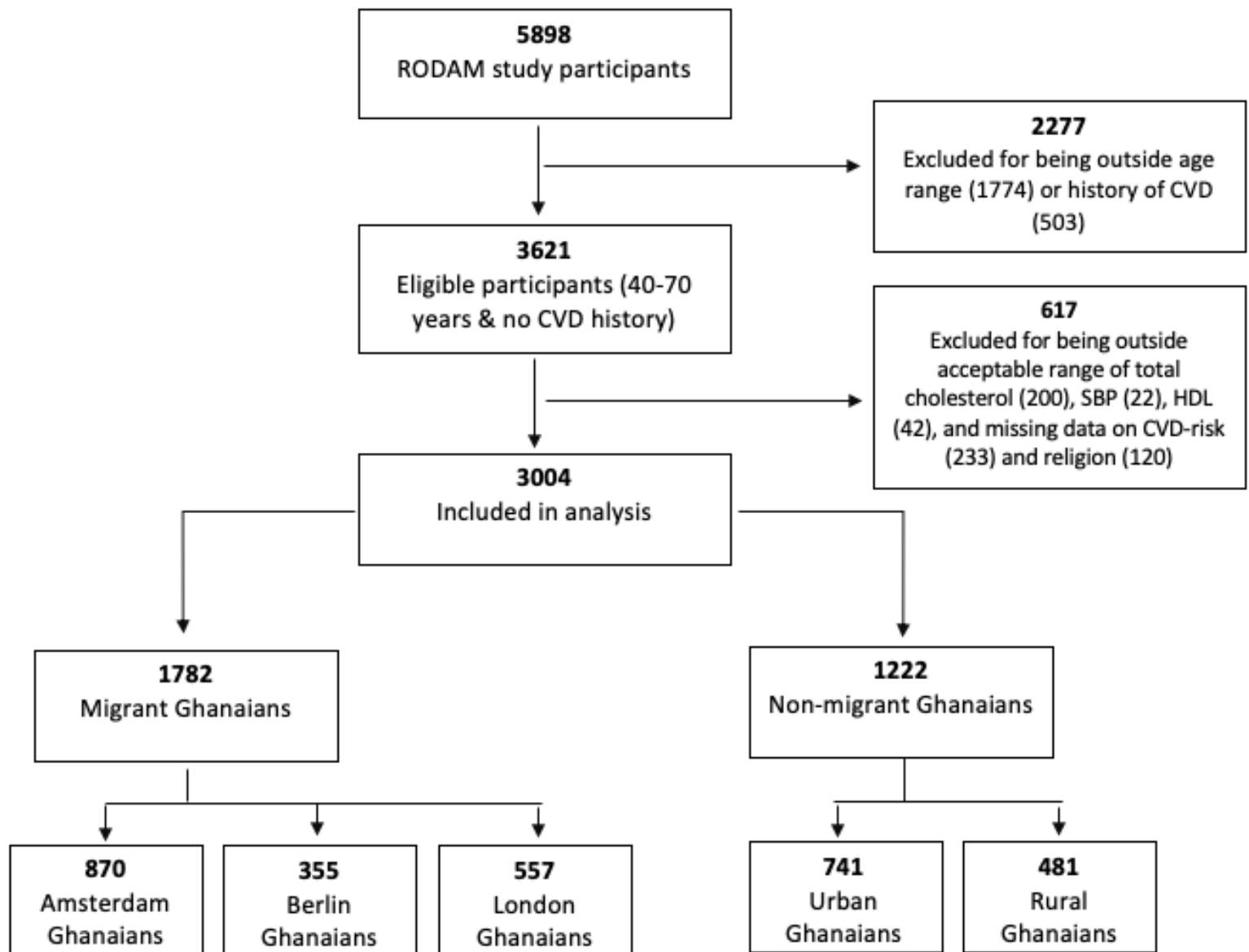

Supplement: Supplementary file 1 [file ijerph-18-02451-s001.pdf]
